# OxyR Is a Convergent Target for Mutations Acquired during Adaptation to Oxidative Stress-Prone Metabolic States

**DOI:** 10.1093/molbev/msz251

**Published:** 2019-10-25

**Authors:** Amitesh Anand, Ke Chen, Edward Catoiu, Anand V Sastry, Connor A Olson, Troy E Sandberg, Yara Seif, Sibei Xu, Richard Szubin, Laurence Yang, Adam M Feist, Bernhard O Palsson

**Affiliations:** 1 Department of Bioengineering, University of California, San Diego, La Jolla, CA; 2 Novo Nordisk Foundation Center for Biosustainability, Technical University of Denmark, Kemitorvet, Kongens, Lyngby, Denmark

**Keywords:** adaptive laboratory evolution, oxidative stress, systems biology

## Abstract

Oxidative stress is concomitant with aerobic metabolism. Thus, bacterial genomes encode elaborate mechanisms to achieve redox homeostasis. Here we report that the peroxide-sensing transcription factor, *oxyR*, is a common mutational target using bacterial species belonging to two genera, *Escherichia coli* and *Vibrio natriegens*, in separate growth conditions implemented during laboratory evolution. The mutations clustered in the redox active site, dimer interface, and flexible redox loop of the protein. These mutations favor the oxidized conformation of OxyR that results in constitutive expression of the genes it regulates. Independent component analysis of the transcriptome revealed that the constitutive activity of OxyR reduces DNA damage from reactive oxygen species, as inferred from the activity of the SOS response regulator LexA. This adaptation to peroxide stress came at a cost of lower growth, as revealed by calculations of proteome allocation using genome-scale models of metabolism and macromolecular expression. Further, identification of similar sequence changes in natural isolates of *E. coli* indicates that adaptation to oxidative stress through genetic changes in *oxyR* can be a common occurrence.

## Introduction

Oxidative stress response is central to microbial adaptability and pathogenicity (Riboulet et al. 2007). Apart from endogenously generated reactive oxygen species (ROS), an array of exogenous factors like environmental conditions, host responses, and antimicrobials trigger reactions that elevate ROS levels. An elaborate ROS defense system enables survival in a multitude of environmental conditions (Wu et al. 2012). This defense machinery ranges from enzymes involved in detoxification of reactive radicals to repair enzymes restoring cellular physiology.

Cellular redox homeostasis is maintained by the activities of ROS-inducible transcriptional regulators controlling the expression of the aforementioned enzyme classes (Seo et al. 2015). OxyR is a widely conserved peroxide-sensing transcriptional regulator in bacteria (Chiang and Schellhorn 2012). It functions as a redox switch and regulates expression of a set of enzymes responsible for the mitigation of ROS-mediated damage. We observed mutations in the *oxyR* gene during the adaptive laboratory evolution (ALE) of two bacterial species, *Escherichia coli* and *Vibrio natriegens*. The *E. coli* evolution was performed in an iron-replete condition that can trigger oxidative stress. A system-level examination revealed improved peroxide stress mitigation ability and reduced DNA damage in the iron-evolved strains. Previously, a constitutive OxyR activation in *E. coli* has been observed by an epigenetic mechanism (Gundlach and Winter 2014); here, we observed direct genetic changes in the *oxyR* sequence resulting in its constitutive activity. Further, the higher growth rate of *V. natriegens*, and thus greater aerobic metabolic flux, also resulted in the acquisition of *oxyR* mutations enabling elevated peroxide tolerance. We performed a 3D amino acid proximity analysis, which revealed the structural impact of the mutations in favor of the oxidized conformation of OxyR, thereby activating expression of ROS-quenching enzymes. Calculations using a genome-scale model of metabolism and macromolecular expression (ME-model) estimated the proteomic cost of the constitutive expression of OxyR-regulated genes that revealed an underlying “fear–greed” trade-off (Utrilla et al. 2016). The identification of similar genetic changes in sequenced natural isolates suggests adaptive targeting of OxyR in diverse environmental conditions.

## Results and Discussion

### ALE in Iron-Replete Environment

Iron homeostasis is fundamental for healthy microbial physiology given the many metabolic processes that depend on enzyme complexes requiring iron as a cofactor. However, an iron overload can be toxic due to the Fenton reaction generating reactive hydroxyl radicals (Imlay et al. 1988; Zheng et al. 1999). Previously, we evolved a genome-edited strain of *E. coli* in the presence of extra iron supplements (Anand et al. 2019). Both independent replicates acquired mutations in *oxyR* during this laboratory evolution.

**Fig. 1.**
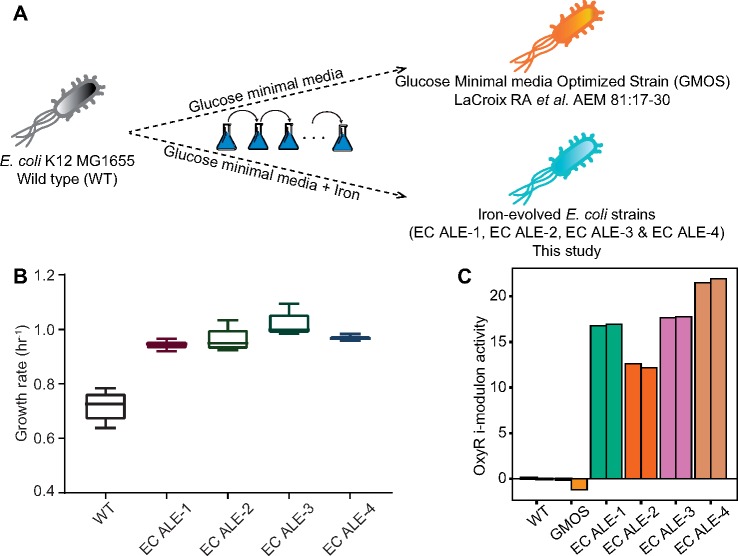
Experimental evolution of *Escherichia coli* in iron-replete conditions. (*A*) The schematic describing the study. (*B*) Growth rate of *E. coli* strains evolved for ∼4 × 10^11^ cumulative number of individual cell divisions in glucose minimal media supplemented with ferrous sulfate and sodium citrate. (*C*) Activity of the OxyR i-modulon estimated by ICA. The bars with identical colors in ICA plot represent biological replicates of the corresponding strain.

To investigate the occurrence of *oxyR* mutations in high iron conditions, we performed additional ALE of wild type (WT) *E. coli* in iron-supplemented M9 medium (fig. 1*A*). Iron supplementation had a growth-promoting effect—all four replicates reached equivalent growth rates as the nonsupplemented control evolutions, but on a shorter adaptive timescale (fig. 1*B*) (LaCroix et al. 2015). We performed whole-genome resequencing of the evolved strains to identify the genetic basis of adaptation. All four evolved replicates had point mutations in *oxyR*. The acquisition of mutation in the same gene in all the strains evolved in an iron-replete condition suggested an adaptive role of these mutations (supplementary table 1, Supplementary Material online). Interestingly, one of the replicates had cysteine at position 208 mutated to tyrosine. This cysteine is reported to be involved in intramolecular disulfide bond formation whose involvement in OxyR regulon activation has been a subject of controversy (Helmann 2002).

### OxyR Mutations Lead to Well-Defined Transcriptomic Changes

The mutation of *oxyR* in iron evolution experiments motivated us to explore the related metabolic consequences. Notably, a random mutagenesis experiment exploring the activation ability of nucleotide changes of the *oxyR* gene identified seven different mutations which resulted in higher *oxyS* (a small regulatory RNA involved in the oxidative stress response) promoter activity (Kullik et al. 1995). In *Xanthomonas campestris*, a nucleotide change in *oxyR* has been reported to impart higher peroxide tolerance (Mongkolsuk et al. 2000). Constitutive expression of the OxyR regulon in *E. coli* has been reported due to epigenetic change, with no genetic change identified (Gundlach and Winter 2014). In order to examine the status of OxyR gene network in the iron-evolved strains, we performed transcriptome analysis of these strains.

We delineated the transcriptional responses using an unsupervised signal deconvolution algorithm, independent component analysis (ICA) (Sastry et al. 2019), which produces a set of independently modulated groups of genes (i-modulons). I-modulons exhibit a significant overlap with the classical regulon definition for a specific transcriptional regulator, except ICA identifies specifically which genes of the regulon respond coherently to stimuli. Further, the ICA decomposition of transcriptomic data quantifies the condition-dependent activity level of each i-modulon, which indicates the activity of its linked transcriptional regulator.

**Fig. 2.**
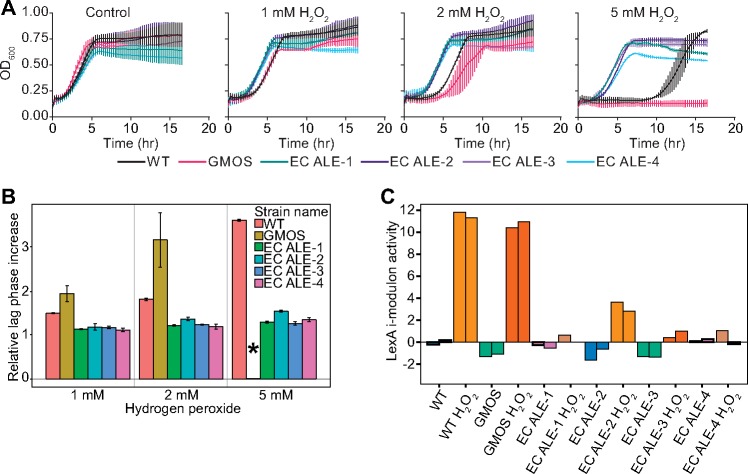
Adaptive impact of *oxyR* mutations. (*A*) Growth profile in the presence of hydrogen peroxide. (*B*) Estimation of growth retardation due to addition of peroxide by calculating the relative increase in the lag phase. * indicates the missing bar corresponding to GMOS strain, as there was no detectable growth at 5 mM peroxide concentration. (*C*) Activity of the LexA i-modulon estimated by ICA. The bars with identical colors in ICA plot represent biological replicates of the corresponding strain.

A specific i-modulon for OxyR has been identified (Sastry et al. 2019). ICA showed a significant activity change in the OxyR i-modulon in iron-evolved strains compared with the WT strain and glucose minimal media-optimized strain (GMOS). All iron-evolved strains showed very high OxyR i-modulon activity (fig. 1*C* and supplementary fig. 1, Supplementary Material online), indicating an increased expression level of the genes in the OxyR i-modulon. This result suggested a constitutive activation of OxyR in the evolved strains. The OxyR-regulated genes mount both protective and preventive responses. Although ROS-scavenging enzymes quench the reactive radicals, Fur and Dps regulate cytosolic iron concentration to limit hydroxyl radical generation (Imlay 2008; Chiancone and Ceci 2010; Frawley and Fang 2014). The activity change was specific to peroxide sensing, as no activity change was observed in the SoxS i-modulon that is related to superoxide sensing (supplementary fig. 2, Supplementary Material online) (Demple and Amabile-Cuevas 1991; Storz and Imlay 1999). Therefore, we examined the effects of peroxide exposure on the evolved strains. We observed a dose-dependent growth retardation in WT and GMOS strains but saw no significant impact of hydrogen peroxide on the growth profile of the iron-evolved strains (fig. 2*A*). Constitutive expression of OxyR-regulated ROS-detoxifying genes rendered them tolerant to peroxide. Potentially, the increased catalase activity in the evolved strains catalyzed a more efficient disproportionation of peroxide which might be responsible for the relatively short lag phase duration (Ma and Eaton 1992; Mishra and Imlay 2012).

DNA exposure to ROS can lead to genomic lesions (Jena 2012). *Escherichia* *coli* has an elaborate SOS response system to respond to DNA damage under ROS stress. This LexA-regulated response ranges from stalling cell division to translesional DNA polymerization and damage repair (Goodman and Woodgate 2013; Imlay 2013; Polosina 2014). We estimated the relative change in the lag phase upon peroxide treatment. The exposure to hydrogen peroxide increased the lag phase duration of WT and GMOS strains in a dose-dependent manner, whereas iron-evolved strains were relatively unaffected (fig. 2*B*). We thus performed mRNA sequencing of the strains after peroxide treatment.

An i-modulon linked to LexA has been discovered (Sastry et al. 2019). LexA is the repressor of the cellular SOS response system to DNA damage (Foti et al. 2010). We observed a high activity of the LexA i-modulon in WT and GMOS strains cultivated in the presence of hydrogen peroxide (fig. 2*C*). Peroxide-generated oxidative stress results in genomic lesions and cells respond to this damage by activating the SOS system (Imlay 2013). The derepression of LexA-regulated genes in WT and GMOS strains explains the extended lag phase observed for these strains upon peroxide treatment. However, the iron-evolved strains did not show a significant increase in the LexA i-modulon activity (fig. 2*C* and supplementary fig. 3, Supplementary Material online). This result suggests less DNA damage in the iron-evolved strains, and it potentially explains the reduced impact of peroxide exposure on the lag phase of these strains.

### Trade-off between Oxidative Stress Tolerance and Adaptation to Higher Growth Rate

The *E. coli* strain evolved to a higher growth rate by optimizing on glucose minimal media, GMOS, showed the highest sensitivity to peroxide stress, with no detectable growth at 5 mM H_2_O_2_ concentration (fig. 2*A* and *B*). We hypothesize that the higher sensitivity is due to proteome reallocation that shifts resources away from the stress-related functions to the growth-promoting processes (Pirt 1982; Utrilla et al. 2016). We support this “fear-versus-greed” trade-off (Utrilla et al. 2016) by showing additional evidence: 1) Higher expression of the peroxide stress-tolerance proteome leads to lower growth in the stress-free environment; and 2) the fast-growing bacterial species *V. natriegens* is very sensitive to peroxide stress in the absence of adaptive genetic changes.

**Fig. 3.**
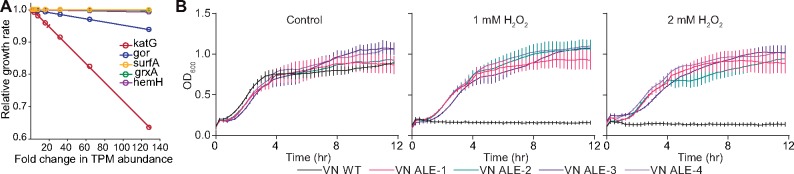
The “fear–greed” trade-off. (*A*) The relative decrease in growth rate upon increase in the expression of OxyR-regulated genes as compared with the expression levels in GMOS. (*B*) Estimation of peroxide sensitivity of the WT and evolved strains of *Vibrio natriegens*.

To evaluate the amount of growth rate reduction due to increased expression of the oxidative-stress-related genes, we simulated the cost of increased protein expression using a genome-scale model of metabolism and protein expression enhanced by a protein-folding network, FoldME (Chen et al. 2017). We found two linear correlations that nicely describe the relationship between proteome cost and reduction in the overall growth rate. First, growth rate decreases as the expression of any single gene in the OxyR regulon increases (fig. 3*A*). Second, the more abundant a gene is in the reference transcriptome, the more significantly it can affect the growth rate (the larger the slope is in fig. 3*A* and supplementary fig. 4, Supplementary Material online). These two simulated scaling laws for the proteome cost allowed us to estimate the growth rate reduction caused by higher expression of the OxyR-regulated genes. According to the transcriptomic data, increased expression of the alkyl hydroperoxide reductase (*ahpC* and *ahpF*) and the catalase/hydroperoxidase *katG* has the largest effect on growth rate. Thus, fast-growing GMOS has limited resources available for activating peroxide stress-tolerance machinery which explains the higher sensitivity of this strain.

Second, to further examine adaptive responses of the trade-off between growth rate and oxidative stress tolerance, we performed ALE of *V. natriegens*, one of the fastest-growing bacteria known (Lee et al. 2016; Panko 2016). However, even after more than 3 × 10^12^ cumulative cell divisions (∼1,000 generations), we did not observe a significant increase in the growth rate (supplementary fig. 5, Supplementary Material online). We added more replicates to further examine potential growth improvements but obtained a similar result. However, adaptive changes in metabolic networks following ALE have previously been observed despite insignificant improvements in growth rate (McCloskey et al. 2018). Therefore, we performed whole-genome resequencing of the evolved strains. We observed that all the evolved replicates of *V. natriegens* (VN ALEs) had acquired one-point mutation each in *oxyR* (supplementary table 2, Supplementary Material online). This motivated us to examine the peroxide tolerance of these evolved *V. natriegens* strains. The *V. natriegens* wild type (VN WT) strain was found to be very sensitive to peroxide treatment, and complete growth retardation was observed at even 1 mM H_2_O_2_. In contrast, the evolved strains were highly tolerant of hydrogen peroxide (fig. 3*B*). Thus, *oxyR* mutations in *V. natriegens* exhibit peroxide tolerance similar to what was seen in *E. coli*. This further supported the notion that proteome reallocation enables better defense to oxidative stress.

### Potential Structural Implication of OxyR Mutations

OxyR functions in vivo as a dimer of dimers and undergoes structural rearrangements in oxidizing and reducing environments (Choi et al. 2001). Both of these redox conformers of OxyR can bind to the target gene promoters but with distinct DNA footprints. Notably, the reduction of OxyR is significantly slower than oxidation and, if the reduced form stability is compromised, even an interaction with RNA polymerase can oxidize the reduced conformer (Choi et al. 2001). Thus, stabilizing the oxidized conformation or destabilizing the reduced conformation may have similar outcome which is activation of OxyR gene regulatory network. We, therefore, used available structural and biochemical information about the OxyR amino acid residues to contextualize the potential impact of mutations observed in the evolved strains by performing amino acid proximity mapping and structural modeling (Choi et al. 2001).

**Fig. 4.**
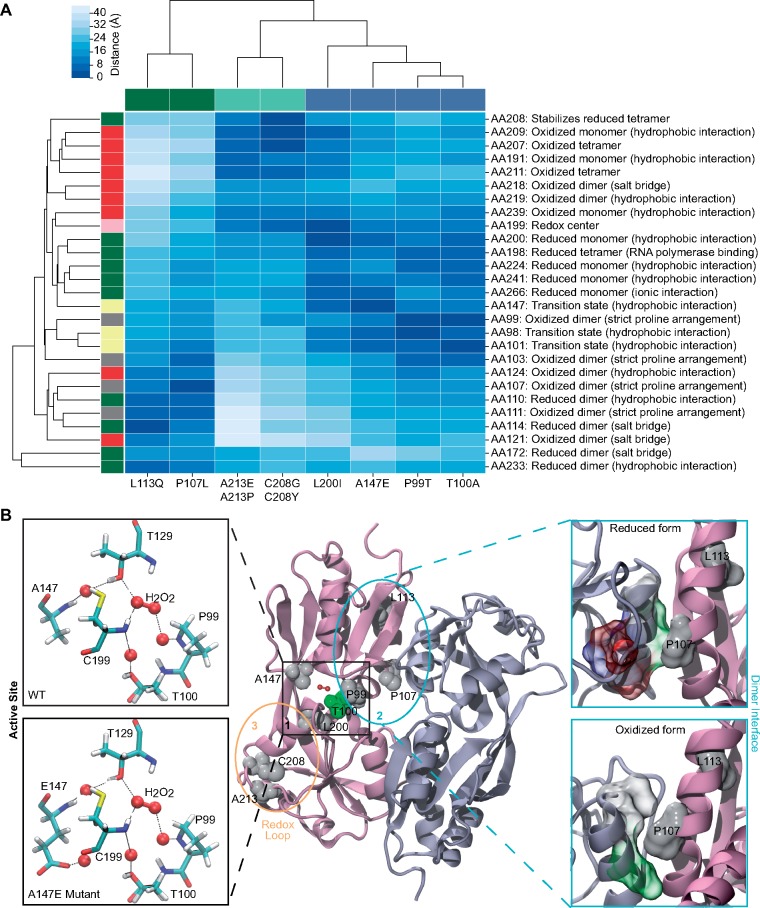
Potential structural impact of OxyR mutations. (*A*) Proximity of OxyR mutants to regions of OxyR structural stability. Distance (Å) between OxyR mutants (columns)—*Escherichia coli* (green), *Vibrio natriegens* (blue), and both (teal)—and residues involved in OxyR stability (rows) is shown. Residues stabilizing reduced OxyR (green); stabilizing oxidized OxyR (red); involved in the monomeric redox transition state (yellow); containing unique proline arrangement (gray) are shown, as well as redox center Cys-199 (pink). (*B*) Mapping of the mutations on the OxyR structure. In the middle panel, two regulatory domains of OxyR that form the dimer are shown in cartoon representation, and colored in mauve and ice-blue, respectively. Mutated residues are shown in space-filling spheres, colored by property of the amino acid side chains (white for hydrophobic side chains, green for polar side chains, blue for positively charged side chains, and red for negatively charged side chains). Three hot spots for mutations are highlighted. In the left panel, residues that form the binding environment of H_2_O_2_, including C199, T129, A147 (E147), P99, and T100 are shown in licorice and colored by atom type. Potential hydrogen bonds between key residues of OxyR, H_2_O_2_, and water molecules (red sphere) are shown by dashed lines. In the right panel, conformational change between α-helices αA and αD on the dimer interface causing the change in the binding environment of P107 is shown. Residues involved in the interface interaction are shown in space-filling volume and colored by amino acid properties as described above.

We used the protein structures of the oxidized and reduced forms of OxyR (PDB: 1I6A and 1I69, respectively) to determine the proximity of amino acid substitutions to known important structural regions (supplementary table 3, Supplementary Material online). As the OxyR amino acid sequences of *E. coli* and *V. natriegens* are ∼77% similar (∼62% identical), and all the observed amino acid substitutions from *V. natriegens* evolution are conserved between the two species (supplementary fig. 6, Supplementary Material online), we also used the structural information of *E. coli* OxyR to explain the potential effect of mutations in *V. natriegens*. We started with showing the consistency between proximity-based analysis of the effect of previously published mutations with known OxyR activity (supplementary fig. 7 and supplementary table 4, Supplementary Material online) and then proceeded with using this framework to interpret the outcomes of the evolved strains (supplementary tables 1 and 2, Supplementary Material online and fig. 4*A*).

The flexible loop region (205–216) dictates transition of redox-active Cys-199 to an outward conformation, and we hypothesize that the reduced flexibility of the loop region impairs the ability of oxidized OxyR to transition back into the reduced form in A213P and A213E mutants. Also favoring the transition state to oxidized OxyR monomer is the hydrophobic environment containing Ala-147. We modeled the atomic interactions facilitated by the glutamic acid (E) substitution at residue 147 to examine the impact of A147E mutation and observed an enhanced H-bonding network that can shift the equilibrium toward the oxidized form (fig. 4*B*). Similarly, we propose that the T100A mutant confers additional hydrophobicity to the transition region which favors the oxidized OxyR monomer. Dimerization of oxidized OxyR requires a unique arrangement of prolines (Pro-99, 103, 107, 111) to form a relatively flat helix surface (Choi et al. 2001). We suggest that mutants P99T and P107L support the dimerization of oxidized OxyR by alleviating the requirement of this unique helix arrangement.

The reduced OxyR monomer is stabilized by the hydrophobic core containing Leu-200. We believe that the hydrophobicity of this core is compromised in L200I mutant, destabilizing the reduced monomeric OxyR. However, the similarities in the chemical properties of Leu and Ile limit the scope of this interpretation and warrant further structural and/or biochemical investigation. Similarly, the redox loop 199–208 is suggested to be required for the tetramerization of reduced OxyR. The conserved residue Cys-208 was found mutated in one of the iron-evolved *E. coli* strains and two of the *V. natriegens*-evolved strains. In an earlier study, the C208Y mutation in OxyR has been reported to result in a similar phenotype (Kullik et al. 1995). Thus, we postulate that C208Y and C208G mutants demonstrate constitutive activity of OxyR due to the destabilization of tetrameric OxyR in its reduced form. A salt-bridge formed between Asp-172 and His-114 stabilizes the reduced OxyR dimer. We suggest that the moderately radical L113Q mutant disrupts the alignment of the A172–H114 salt-bridge and destabilizes dimerization in the reduced form.

### OxyR Alleles in Natural Environment

Microbes are exposed to diverse stress conditions in natural environments, and their survival requires adaptation to the ambient environment. Oxidative stress is a key challenge experienced by microbes in nature, either due to biotic or abiotic factors (Imlay 2019). We therefore explored the possibility of adaptive *oxyR* mutations in *E. coli* strains isolated from natural environments. On the Pathosystems Resource Integration Center (PATRIC) (Wattam et al. 2017), OxyR sequences for 17,253 unique *E. coli* genomes were available. Of these, there exist 757 unique DNA sequences coding for 314 unique amino acid sequences of OxyR. We compared these sequences with the sequences recognized to cause constitutive activation of OxyR as reference to screen for genomes with similar genetic changes (supplementary table 5, Supplementary Material online).

**Fig. 5.**
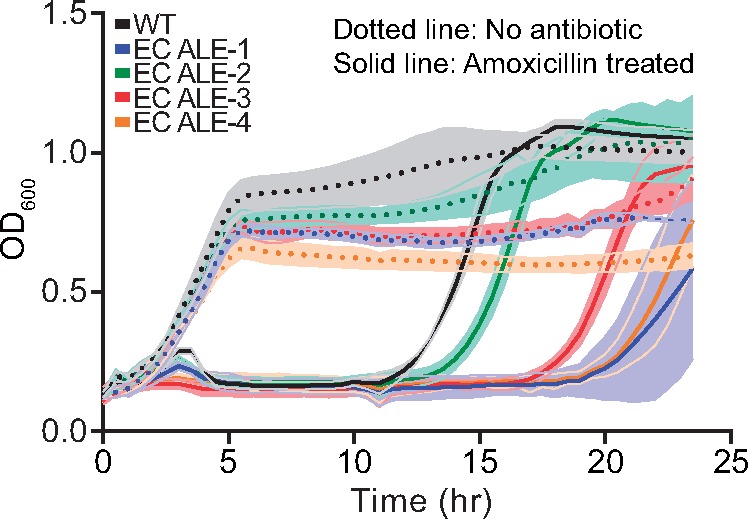
Amoxicillin sensitivity of the iron-evolved strains. Width of shaded bands represents standard deviation of the corresponding growth trajectory.

Seven genomes (table 1) were identified to have sequence changes that may activate OxyR constitutively, of which two had the same amino acid substitutions. Interestingly, there was a conflicting report on the susceptibility toward amoxicillin for these genomes. As amoxicillin treatment has been reported to result in higher oxidative stress (Hoeksema et al. 2018), we performed the amoxicillin sensitivity assay for the iron-evolved strains. Three out of the four strains showed an extended lag phase upon amoxicillin treatment (fig. 5). This ruled out any resistance-conferring abilities for these mutations and suggests either increased or similar sensitivity to this antibiotic in these strains as compared with the WT cells. This result further reveals a potential trade-off of constitutive expression of OxyR gene regulatory network.

**Table 1.** *Escherichia coli* Natural Isolates Showing OxyR Sequence Change Similar to the Mutations Resulting in Constitutive Activation of the OxyR.

Genome ID	OxyR Sequence Change	AMR Information
1280958.3	R201C	N.A.
562.23930	R201C	Amoxicillin resistant
562.23771	R201S	Amoxicillin susceptible
562.10507	A213T	N.A.
562.31230	P107T	N.A.
562.22907	A147V	Amoxicillin susceptible
562.23860	L200V	Amoxicillin resistant

note.—Antimicrobial resistance (AMR) information has been taken from PATRIC. (N.A. stands for “not available”).

## Conclusions

Several physiological perturbations may have convergent downstream impact, thus requiring a similar response mechanism. Here, we observed that metabolic states resulting from adaptation to peroxide stress come with a gain of function mutation in *oxyR*. Interestingly, the stress caused by the iron overload did not target any gene directly involved in the cellular iron homeostasis. The iron toxicity mitigation abilities provided by the *oxyR* mutation may have relieved the selection pressure on other genes. The mutations favored the oxidized conformation of OxyR that activates its regulatory network. OxyR is a positive regulator of peroxide stress mitigation enzymes; however there are reports of dual regulation of several target genes by reduced and oxidized conformers (Loprasert et al. 2000; Ieva et al. 2008). In these studies, the expression of catalase and alkyl hydroperoxide reductase is reported to be tightly controlled by the redox state of OxyR; reduced and oxidized forms acting as repressor and activator, respectively. Therefore, structurally locked conformation provided better protection against peroxide than redox switching OxyR potentially due to absence of any repression by the reduced conformer and absence of response time lag (Loprasert et al. 2000; Seaver and Imlay 2001; Geisel 2011). However, this fitness improvement comes at a cost of reduced growth phenotype. This trade-off due to constrained proteome and energy allocation shapes the optimal response and adaptabilities of the organism in an environmental condition (Utrilla et al. 2016).

The presence of constitutively active OxyR alleles in natural isolates demonstrates the adaptive significance of this change in regulatory structure. A large-scale involvement of oxidative stress in shaping microbial physiology requires further examination of these isolates to attribute environmental association to these genetic changes.

## Materials and Methods

Detailed materials and methods used for strain generation, ALE, DNA sequencing, RNA sequencing, i-modulon decomposition, phenotype characterization, structural interpretation, proteome-constrained model simulation, genome-scale computations, and allelic variant search are provided as Supplementary Material online.

## Supplementary Material

msz251_Supplementary_Data

## Notes

The whole-genome sequencing data have been submitted to NCBI Sequence Read Archive (SRA accession: PRJNA559568), and expression profiling data have been deposited at NCBI Gene Expression Omnibus (GEO accession number GSE135516).
