# Supplementary material for: OxyR Is a Convergent Target for Mutations Acquired during Adaptation to Oxidative Stress-Prone Metabolic States
Source: Mol Biol Evol. 2019 Oct 25;37(3):660–7. doi: 10.1093/molbev/msz251 (PMC7038661; doi:10.1093/molbev/msz251)
Supplement: msz251_Supplementary_Data [file msz251_supplementary_data.zip › OxyR_supplementary_file.pdf]

Supplementary material for:

OxyR is a convergent target for mutations acquired during adaptation to oxidative stress-prone metabolic states

Anand *et al.*

Bernhard O. Palsson.
E-mail: palsson@ucsd.edu

Contents:

Methods

- Materials
- Adaptive laboratory evolution (ALE) and DNA resequencing
- Transcriptomics
- I-modulon decomposition
- Lag phase estimation
- ME Model simulation
- Structural interpretation of OxyR mutations
- Search for constitutively active OxyR allele

Data Availability

Supplementary Figures

Supplementary Tables

Supplementary References

METHODS

Materials

Escherichia coli K-12 MG1655 (ATCC 700926) and *Vibrio natriegens* (ATCC 14048) were used as the wild type strains. Bioscreen C Reader system was used for the growth profiling. Media components were purchased from Sigma-Aldrich (St. Louis, MO). Hydrogen peroxide was purchased from Fisher scientific (H325).

Adaptive laboratory evolution (ALE) and DNA resequencing

ALE was performed as previously described (1). Cultures were serially propagated at 37°C and well-mixed for proper aeration using an automated system that passed the cultures to fresh flasks once they had reached an OD_{600} of 0.3 (Tecan Sunrise plate reader). *E. coli* evolution experiment was performed on M9 minimal medium containing 4 g/L glucose supplemented with 20 μM $FeSO_4$ and 10 mM sodium citrate. *V. natriegens* evolution experiment was performed on M9 minimal medium containing 4 g/L glucose with 1.5% NaCl added to the media. *E. coli* and *V. natriegens* evolution experiments were performed with four and ten biologically independent replicates respectively (Suppl. Table 6). The slope of $\ln(OD_{600})$ vs. time of four OD_{600} measurements from each flask was used to determine the growth rate. DNA resequencing was performed on a clone from the end points of evolved strains as described earlier (1). The *E. coli* sequencing reads were mapped to NC_000913.3 and *V. natriegens* sequencing reads were mapped to CP009977.1 (chromosome 1) as well as CP009978.1 (chromosome 2) to identify mutations.

Transcriptomics

RNAseq was performed using two biological replicates. The strains were grown in a condition the same as that used during ALE in M9 minimal media. 2 mM hydrogen peroxide was used for preparing peroxide stressed cell samples. Total RNA isolation, rRNA removal and

sequencing library preparation was performed as previously described except RNA HyperPrep Kit (KK8541) was used instead of the KAPA Stranded RNA-Seq Kit (Kapa Biosystems KK8401) (1). Libraries were ran on a NextSeq (illumina). Expression profiling was performed as previously described (2). Raw sequencing reads were mapped to the reference genome (NC_000913.3) using bowtie v1.1.2 (3) with a maximum insert size of 1000 and two maximum mismatches after trimming 3 bp at the 3' ends. Transcript abundance was quantified using summarizeOverlaps from the R GenomicAlignments package, with strand inversion for the dUTP protocol and strict intersection mode (4). We then estimated the dispersion and differential expression level of each gene using DESeq2 (5). Transcripts per Million (TPM) were calculated by DESeq2. RNA-seq data for paraquat treatment (250 μM) was obtained from GSE65711.

I-modulon decomposition

The expression profiles generated in this study were combined with a collection of 278 *E. coli* expression profiles previously generated in our research group. Independent component analysis (ICA) was performed as described by Sastry *et al.* 2019 (6). Briefly, the WT MG1655 expression profile reported in this manuscript was used as the baseline condition to center the expression compendium. Then, we executed FastICA 100 times with random seeds and a convergence tolerance of 10^{-7}. The number of components in each iteration was constrained to the number of components that could reconstruct 99% of the variance as calculated by principal component analysis. The resulting components were clustered using DBSCAN to identify robust independent components. I-modulons were extracted from independent components by iteratively removing genes with the largest absolute value and computing the D'agostino K^2 test statistic (7) of the resulting distribution. Once the test statistic fell below a cutoff of 500 (identified through a sensitivity analysis (6)), we designated the removed genes an i-modulon.

Lag time estimation

We fit the absorbance measurements obtained from the reader using non-linear least squares. We used the “nls” command on R to estimate the growth parameters of the Baranyi growth model in each condition (8). The default starting values for lag phase duration, μ_{max} , $\log_{10}(N_0)$ and $\log_{10}(N_{max})$ were initially set to 4, 0.8, 0.1, 0.6. We ran a sensitivity analysis in which we computed the sum of squared errors from fitting the non-linear growth model to the absorbance measurements from time t = 0 to time t = T (with T varying from 2 hours to the total duration of the experiment) to select time thresholds and better starting values for the growth curve parameters in an unbiased fashion. Findpeaks from the Pracma package (9) was used to find the times at which the sum of squared errors minima occur. We then selected the latest time point at which a minimum occurs and used the estimated growth parameters as the starting values for a subsequent nonlinear squares regression run. We report the estimated parameters from the second run.

ME-model evaluation for the growth reduction caused by increased proteome cost

To illustrate the ‘fear-greed tradeoff’, we evaluate the optimal growth of the iron-evolved strains based on the differential expression of the OxyR regulon genes using the FoldME model (10). Fourteen out of the 33 genes in the regulon and i-modulon are currently incorporated into the model. Hence, for a more accurate quantification, the calculation involves two steps: 1) simulate the effect on growth caused by increased expression of individual genes using FoldME; 2) extrapolate the effect to other un-modeled genes and assess the overall reduction of growth.

To simulate *E. coli* growth with the experimentally determined proteome cost of OxyR regulon, we first created an extra reaction representing the total protein mass in the cell:

$$total\_prot\_mass = \sum_i mw_i \cdot v_{translation\_i}$$

where mw_i is the molecular weight, and $v_{translation\_i}$ is the translation flux for protein i. The summation goes over all proteins in the model. Then, we formulated a constraint to specify the mass fraction for each of the 14 modeled OxyR regulon genes:

$$mw_i \cdot v_{translation_i} \geq mf_i \cdot total\_prot\_mass$$

where mf_i is the mass fraction of the gene calculated from the TPM values in the GMOS strain. This simulation computed the optimal growth rate of the GMOS strain that was used as a reference for comparison.

Next, we increased the mass fraction of one gene to $Fc =$ 2, 4, 8, 16, 32, 64, 128 folds of its reference value while keeping the mass fraction of other OxyR regulon genes the same as in the GMOS strain. The optimal growth computed from this set of simulations showed that the growth rate reduction scaled linearly with the fold change (Fc) in expression of individual proteins (Figure 3B). We noticed that the more abundant a protein was in the GMOS strain, the larger effect it would have on the overall growth (the larger the rate of growth reduction, or the steeper the slope in Figure 3B). Data fitting indicated an additional linear relationship between this slope and the mass fraction of the gene in GMOS (Supp. Figure 4).

Structural interpretation of OxyR mutations

Structural regions studied in OxyR have biochemical properties which allow them to induce the redox switch from reduced to oxidized forms of OxyR. Therefore, structural impact of OxyR mutations has been assessed by mapping the proximity of the mutated amino acid residue to the amino acid residues known to be important for the stability of the monomeric, dimeric and tetrameric OxyR in reduced or oxidized conformation. Here, we use the atomic coordinate files (PDB: 1I69 and 1I6A) to determine the minimum point-to-point distances between the $C\beta$ atoms (Cα for glycine) of mutations (ALE endpoints and known mutations) to each structural region.

Seaborn python package was used to cluster the mutations and regions for visualization (Fig. 4A & Supp. Fig. 7). Visualization of the OxyR structure is prepared using the reduced form of *E. coli* OxyR (PDB code: 1I69), and oxidized form of *Corynebacterium glutamicum* OxyR (PDB code: 6G1B).

Search for constitutively active *oxyR* allele

oxyR sequences of all *E. coli* genomes available in Pathosystems Resource Integration Center (PATRIC) were downloaded on May 3rd, 2019. We obtained sequences for 17,253 unique Genome IDs. that were compared for genetic diversity in *oxyR*. The consensus amino acid sequence of OxyR among natural isolates was 100% identical to that of *E. coli* K-12 MG1655. Of these, there exist 757 unique DNA sequences of *oxyR* that code for 314 unique amino acid sequences of OxyR. We performed multiple sequence alignment based on fast fourier transformation using MAFFT online service (MAFFT version 7) (11) and searched for sequence changes listed in supplementary table 5.

SUPPLEMENTARY FIGURES

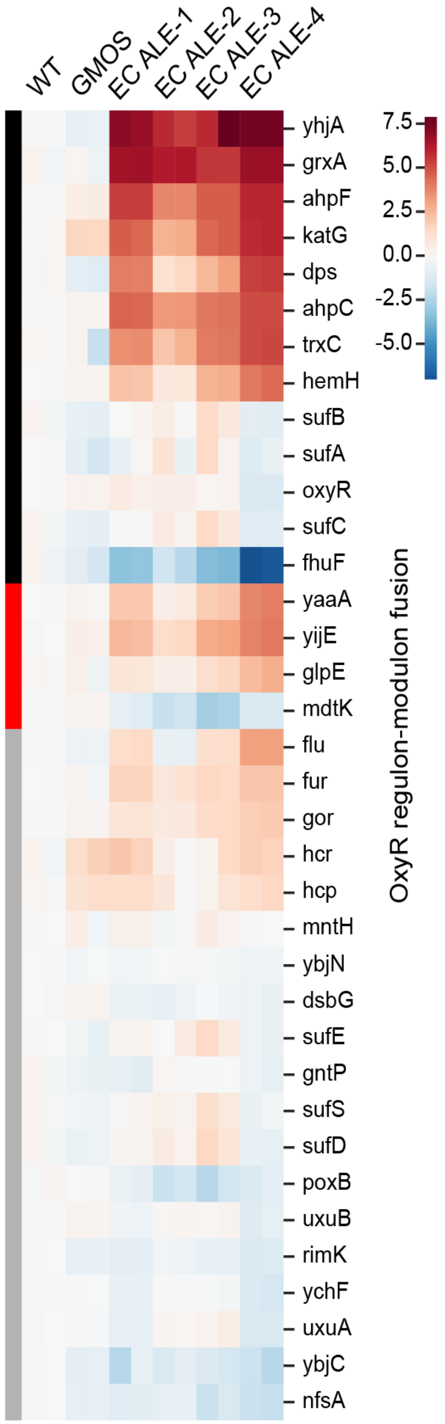

Supplementary Figure 1: Heatmap showing expression of genes belonging to OxyR regulon and i-modulon. Genes grouped under black bar are common to the regulon and i-modulon, whereas those under red and grey bars are specific to i-modulon and regulon respectively.

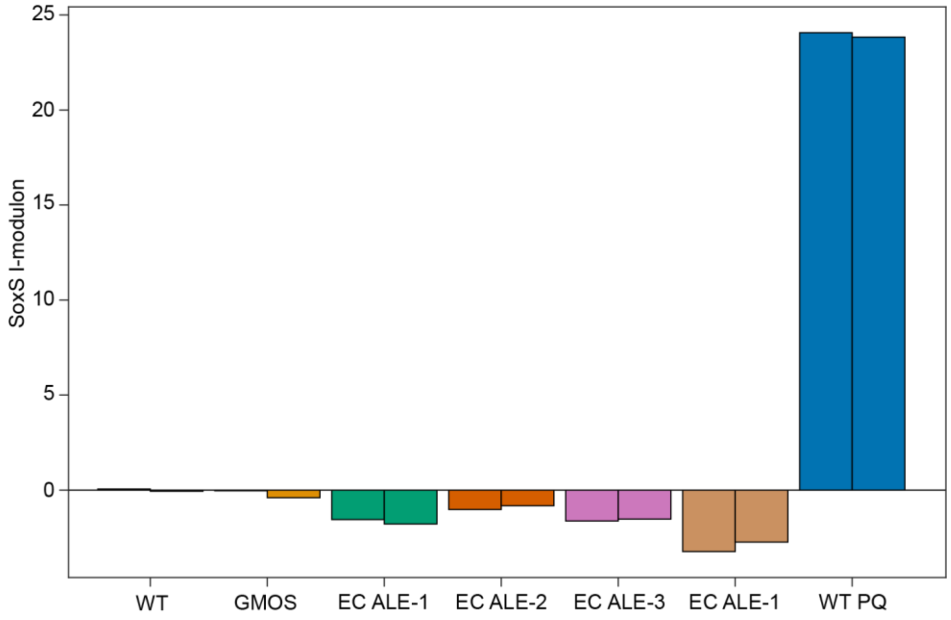

Supplementary Figure 2: Activity of the SoxS i-modulon estimated by independent component analysis (ICA). The bars with identical colors in ICA plots represent biological replicates of the corresponding strain. WT PQ is the positive control showing activation of this i-modulon when *E. coli* is treated with superoxide generating reagent paraquat.

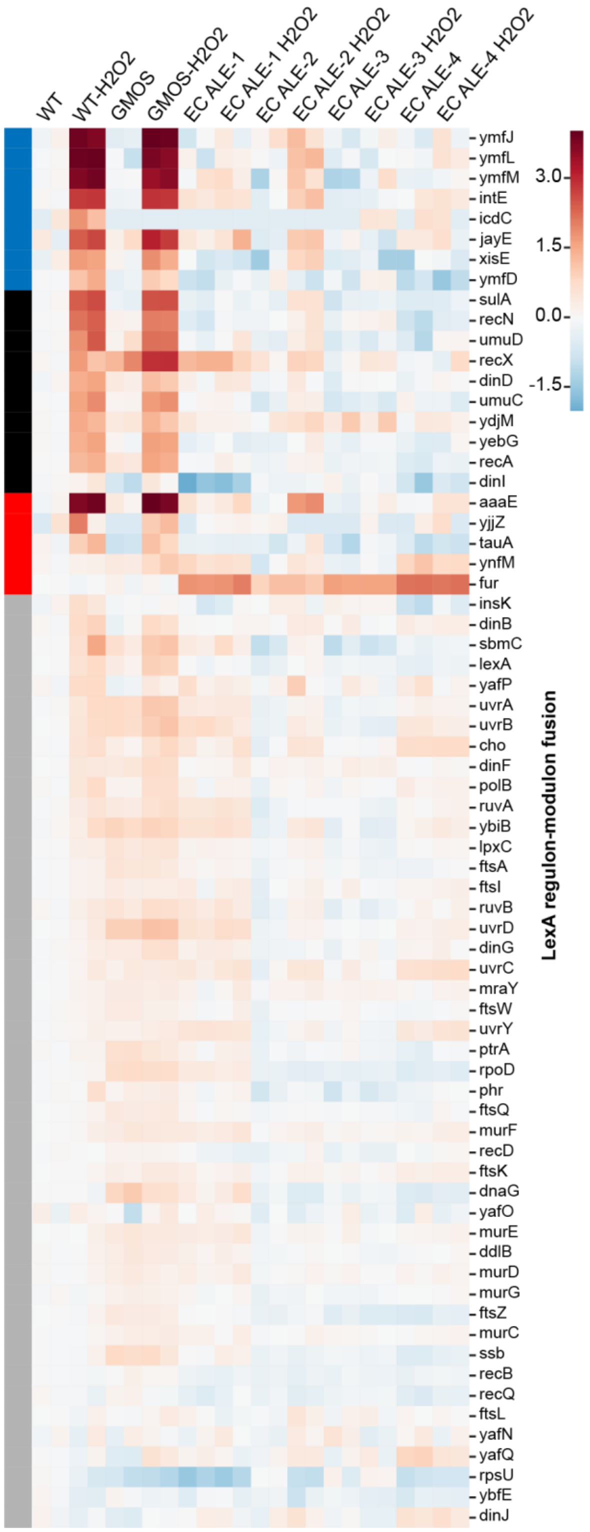

Supplementary Figure 3: Heatmap showing expression of genes belonging to the LexA regulon and i-modulon. Genes grouped under black bar are common to the regulon and i-modulon, whereas those under red and grey bars are specific to i-modulon and regulon respectively. Blue bars groups gene belonging to e14 prophage which is integrated to the genome of *E. coli* K12 MG1655.

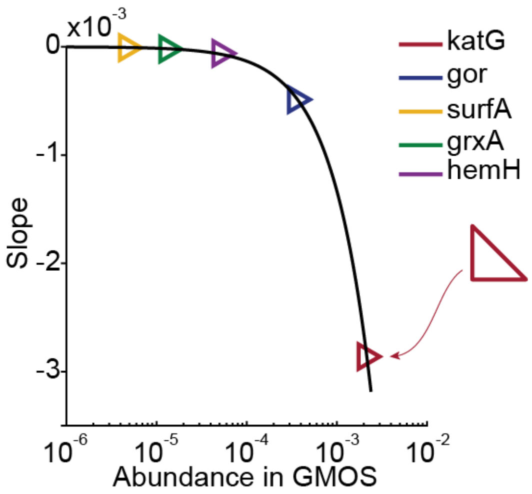

Supplementary Figure 4: The rate of growth rate reduction (the slope in Figure 3B) scales linearly with the abundance of the gene in the GMOS strain. The abundance of gene is measured in mass fraction calculated from the TPM value.

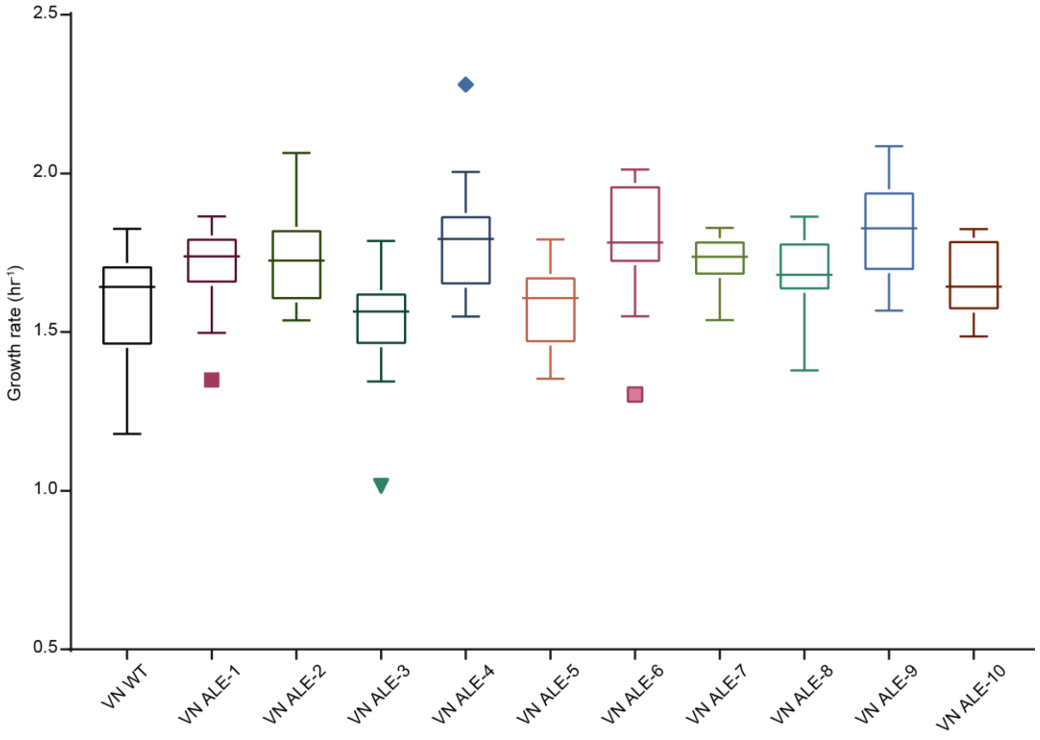

Supplementary Figure 5: Adaptive laboratory evolution of *V. natriegens*. The box plot shows growth rates at the start of the evolution experiment (VN WT) and at the end of the evolution experiment (VN ALE).

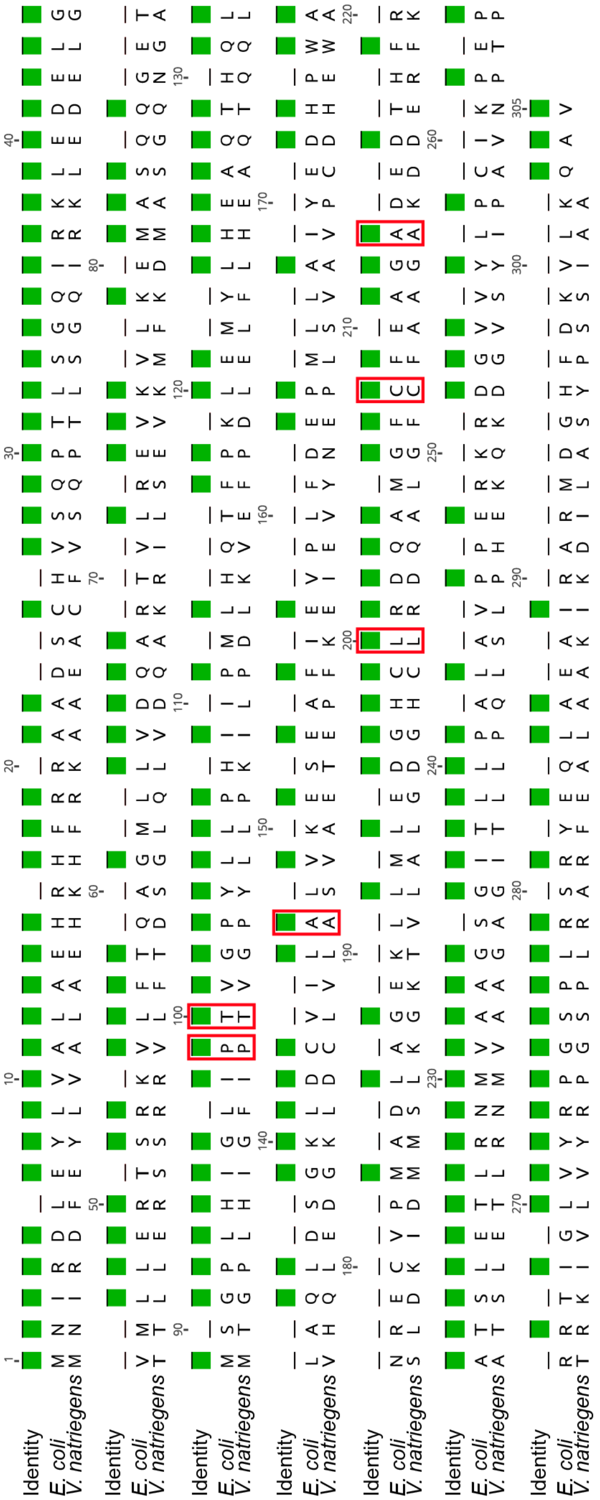

Supplementary Figure 6: Sequence alignment of OxyR sequences from *E. coli* and *V. natriegens*. Green bar indicates sequence identity at the corresponding positions. The mutations observed during *V. natriegens* evolution have been highlighted in red.

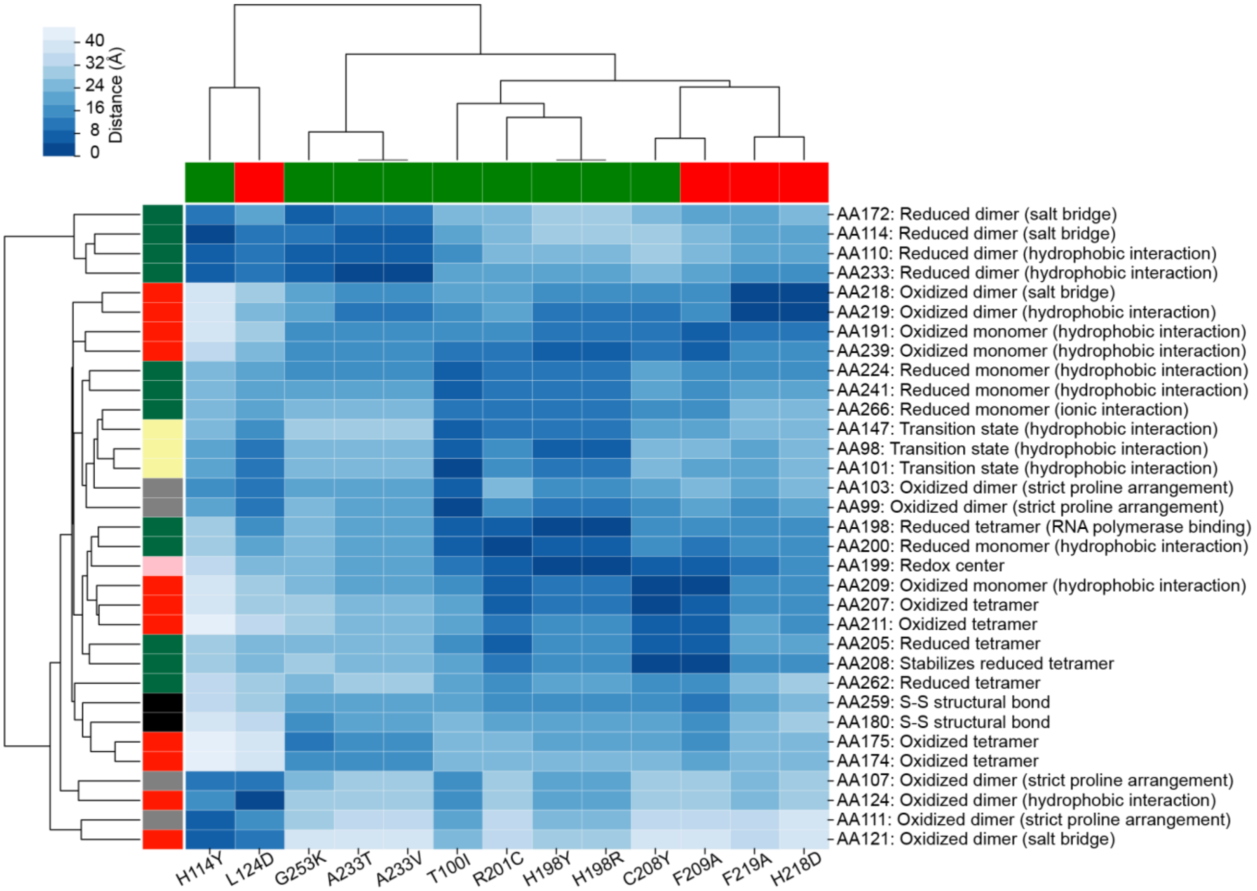

Supplementary Figure 7: Structural impact of known *oxyR* mutations with constitutive activity (green) and decreased activity (red). (A) Proximity of OxyR mutants to regions of OxyR structural stability. Distance (Å) between OxyR mutants (columns) and residues involved in OxyR stability (rows) is shown. Residues stabilizing reduced OxyR (green); stabilizing oxidized OxyR (red); involved in the monomeric redox transition state (yellow); containing unique proline arrangement (grey) are shown, as well as redox center Cys-199 (pink).

SUPPLEMENTARY TABLES

Supplementary Table 1: List of mutations observed in *E. coli* MG1655 strains evolved with iron

**Strain**	**Mutations**					
EC ALE-1	*oxyR* (A213P)	*rpoB*# (I966F)	*rpoC*# (R1075P)			
EC ALE-2	*oxyR* (C208Y)	3,815,859 Δ82 bp rph	*yoeG* (2067837 G→T)			
EC ALE-3	*oxyR* (P107L)	3,815,859 Δ82 bp rph	*yoeG* (2067837 G→T)	betT → / → yahA (331806 G→A)	*ydcC* (W20*)	*yegK* (S14P)
EC ALE-4	*oxyR* (L113Q)	3,815,859 Δ82 bp rph	*gspD* (T578T)	*yheS* (R346H)	*bdcA* (A43S)	

#The mutation frequency of *rpoB* and *rpoC* were 63.6 and 38.4 % respectively.

Supplementary Table 2: List of *oxyR* mutations observed in evolved *V. natriegens* strains

**Strain**	***oxyR* mutation**
VN ALE-1	T100A
VN ALE-2	A147E
VN ALE-3	A213E
VN ALE-4	C208G
VN ALE-5	T100A
VN ALE-6	A147E
VN ALE-7	A213E
VN ALE-8	C208G
VN ALE-9	P99T
VN ALE-10	L200I

Supplementary Table 3: Important residues in oxidized or reduced conformation of OxyR

Stabilization Category	Residue	Description
Redox Center	199	Cys-199 is the redox center of OxyR
Structural S-S Bond	180	Stabilizes tertiary structure
	259	
Reduced Monomer	200	Hydrophobic core which stabilizes monomeric OxyR in its reduced form
	224	
	241	
	266	Ionic interaction with Cys-199 in the reduced monomeric form
Transition State	98	Hydrophobic interactions and limited space force Cys-199 oxidized intermediate out of stabilized pocked
	101	
	147	
Oxidized Monomer	191	Hydrophobic interactions stabilized oxidized Cys-199. Stabilizes monomeric oxidized OxyR
	209	
	239	
Reduced Dimer	110	Forms hydrophobic core to stabilize OxyR dimerization in reduced form. Ile-110 also part of oxidized dimer interface but I100 mutants do not disrupt OxyR activity
	233	
	114	Stabilizes reduced OxyR dimer via salt-bridge interaction
	172	
Oxidized Dimer	99	Strict flat helix proline arrangement needed for oxidized dimer configuration
	103	
	107	
	111	
	124	Forms hydrophobic core to stabilize OxyR dimerization in reduced form. Leu-124 also part of reduced dimer interface but L124 mutants disrupt OxyR activity
	219	
	121	Stabilizes oxidized OxyR dimer via salt-bridge interaction
	218	
Reduced Tetramer	198	Required for stable redox loop (199-208). H198 mutants with large groups disrupt redox loop structure and allow OxyR to bind RNAP / DNA with constitutive activity
	205	Stabilizes reduced tetramer via side-chain interactions. Cys-208 / Cys-199 disulfide bond subject of debate. Can stabilize oxidized monomer, or reduced tetramer.
	262	
	208	
	199-208	
Oxidized Tetramer	174	Stabilizes oxidized tetramer via side-chain interactions.
	175	
	207	
	211	

Supplementary Table 4: Existing mutants and proximity to known important residues

.	Known Mutation	Proximal AA residue(s)	Distance (Å)	Description
Constitutively Active	T100I	99, 101, 98	3.8, 3.8, 5.6	T→ I limits space and increases hydrophobicity of the transition region, favoring out of pocket cys-199
	H114Y	114	0	Reduced dimer destabilized via salt-bridge
	H198Y<br>H198R	198	0	Disruption of redox loop in the reduced tetramer promotes binding of RNAP
	R201C	200, 199	3.8, 5.6	Increased activity of redox center
	A233V<br>A233T	233	0	Decrease of hydrophobicity in hydrophobic core of reduced dimer interaction
	G253K	233, 110	8.1, 8.5	Disruption of hydrophobic interactions involved in dimerization of reduced form
Increase H_2O_2 Sensitivity	L124D	124	0	Disrupts oxidized & reduced dimer
	F209A	209	0	Destabilization of oxidized monomer
	H218D	218	0	Disrupts oxidized dimer formation
	F219A	219	0	Disrupts oxidized dimer formation

Supplementary Table 5: Sequence changes resulting in constitutive activation of OxyR:

Amino acid change	Reference
P99T	VN ALE
T100A/I	VN ALE and (12)
P107L	EC ALE
I110D	(13)
L113Q	EC ALE
H114Y	(12)
A147E	VN ALE
H198Y/R	(12)
L200I	VN ALE
R201C	(12)
C208Y/G	EC ALE, VN ALE and (12)
A213P/E	EC ALE, VN ALE
A233V/T	(12)
G253K	(12)

Supplementary Table 6: Sample code used during running the ALE machine

Sample	ALE code
EC ALE-1	A17F45
EC ALE-2	A18F39
EC ALE-3	A19F39
EC ALE-4	A20F40
VN ALE-1	A2F122
VN ALE-2	A4F191
VN ALE-3	A5F114
VN ALE-4	A7F131
VN ALE-5	A3F126
VN ALE-6	A12F182
VN ALE-7	A15F175
VN ALE-8	A8F196
VN ALE-9	A13F178
VN ALE-10	A16F183

Supplementary References

1. Anand, A., Olson, C.A., Yang, L., Sastry, A.V., Catoiu, E., Choudhary, K.S., Phaneuf, P.V., Sandberg, T.E., Xu, S., Hefner, Y. *et al.* (2019) Pseudogene repair driven by selection pressure applied in experimental evolution. *Nat Microbiol*, **4**, 386-389.
2. Seo, S.W., Kim, D., Latif, H., O'Brien, E.J., Szubin, R. and Palsson, B.O. (2014) Deciphering Fur transcriptional regulatory network highlights its complex role beyond iron metabolism in Escherichia coli. *Nat. Commun.*, **5**, 4910.
3. Langmead, B., Trapnell, C., Pop, M. and Salzberg, S.L. (2009) Ultrafast and memory-efficient alignment of short DNA sequences to the human genome. *Genome Biol.*, **10**, R25.
4. Lawrence, M., Huber, W., Pagès, H., Aboyoun, P., Carlson, M., Gentleman, R., Morgan, M.T. and Carey, V.J. (2013) Software for computing and annotating genomic ranges. *PLoS Comput. Biol.*, **9**, e1003118.
5. Love, M.I., Huber, W. and Anders, S. (2014) Moderated estimation of fold change and dispersion for RNA-seq data with DESeq2. *Genome Biol.*, **15**, 550.
6. Sastry, A.V., Gao, Y., Szubin, R., Hefner, Y., Xu, S., Kim, D., Choudhary, K.S., Yang, L., King, Z.A. and Palsson, B.O. (2019) The Escherichia coli Transcriptome Consists of Independently Regulated Modules. *bioRxiv*.
7. D'Agostino, R.B., Belanger, A. and D'Agostino, R.B. (1990) A Suggestion for Using Powerful and Informative Tests of Normality. *Am. Stat.*, **44**, 316-321.
8. Baranyi, J. and Roberts, T.A. (1994) A dynamic approach to predicting bacterial growth in food. *Int. J. Food Microbiol.*, **23**, 277-294.
9. Borchers, H.W. Practical Numerical Math Functions [R package pracma version 2.2.5].
10. Chen, K., Gao, Y., Mih, N., O'Brien, E.J., Yang, L. and Palsson, B.O. (2017) Thermosensitivity of growth is determined by chaperone-mediated proteome reallocation. *Proc Natl Acad Sci U S A*, **114**, 11548-11553.
11. Katoh, K., Rozewicki, J. and Yamada, K.D. (2017) MAFFT online service: multiple sequence alignment, interactive sequence choice and visualization. *Brief. Bioinform.*
12. Kullik, I., Toledano, M.B., Tartaglia, L.A. and Storz, G. (1995) Mutational analysis of the redox-sensitive transcriptional regulator OxyR: regions important for oxidation and transcriptional activation. *J. Bacteriol.*, **177**, 1275-1284.
13. Choi, H., Kim, S., Mukhopadhyay, P., Cho, S., Woo, J., Storz, G. and Ryu, S.E. (2001) Structural basis of the redox switch in the OxyR transcription factor. *Cell*, **105**, 103-113.
